# Novel recombinant proteins and peptides from *Clonorchis sinensis* and *Opisthorchis viverrini* for liver fluke exposure ELISA

**DOI:** 10.1016/j.bbrep.2023.101516

**Published:** 2023-07-18

**Authors:** Sumathy Mohan, Mohan Natarajan, John G. Bruno

**Affiliations:** a University of Texas Health Science Center at San Antonio, Department of Pathology & Laboratory Medicine, 7703 Floyd Curl Drive, San Antonio, TX, 78229, USA; b Nanohmics Inc., 6201 E. Oltorf Street, Suite 400, Austin, TX, 78741, USA

**Keywords:** *Clonorchis*, *Opisthorchis*, ELISA, Peptide, Recombinant protein, Taiwan, Vietnam

## Abstract

Human serum samples from individuals living in Vietnam and Taiwan suspected of past *Clonorchis sinensis* or *Opisthorchis viverrini* infection were screened using several novel peptides and recombinant liver fluke proteins to determine if any consistent patterns could be discerned and used as the basis for future liver fluke ELISA development. Absorbance values at 405 nm were compared to those of pooled unexposed normal human serum and analyzed for statistical significance. The data exhibited some interesting patterns consistent with egg antigen sequestration in the gut possibly leading to lower serum antibody levels and potential regional exposure differences between Vietnamese and Taiwanese subjects. In particular, antibodies against Cathepsin B and B2 peptides, as well as a partial Cahedrin Domain peptide may be elevated in some Taiwanese serum samples while antibodies against recombinant *Clonorchis* egg protein and Hepatocellular Carcinoma Peptide Antigen 59 may be elevated in some samples from both Taiwan and Vietnam. The data appear to suggest that some of the novel recombinant protein and peptide antigens selected and tested herein warrant further study with larger sample sizes as possible targets for detecting anti-liver fluke antibodies by ELISA from humans suspected of liver fluke infections.

## Highlights

• Six different novel recombinant liver fluke proteins or peptides were screened for exposure ELISA potential.
• Some of the novel targets reacted against human serum samples from Taiwan and Vietnam.
• A recombinant partial Cs44 protein did not react with serum samples perhaps due to a missing or hidden epitope.
• A Clonorchis egg protein did not react strongly with sera perhaps due to egg sequestration in the gut.

## 1 Introduction

Liver fluke infections from consumption of undercooked fish can persist for decades in humans leading to hepatic and bile duct cancer (cholangiocarcinoma), which affects tens of millions of people worldwide especially across Asia, parts of Europe and Russia [1,2]. Numerous ELISA protocols have been published for direct detection of parasite antigens as well as induced antibodies in human sera. For the induced antibody liver fluke exposure ELISAs, some researchers have reported the use of extracted, secreted or shed antigens from adult flukes, but such approaches are prone to being less consistent from batch to batch [[3], [4], [5]]. Other groups have reported the use of recombinant fluke antigens to coat ELISA microwells, which is easier to control from lot to lot, thereby, leading to greater assay consistency [[6], [7], [8], [9], [10]]. In the present work, we report on the use of two recombinant proteins from *C. sinensis* adults and eggs as well as N-terminal peptides originating from *O. viverrini* listed in Table 1 and preliminary assessment of their potential for detection of previous liver fluke exposure using unexposed pooled normal human serum (NHS) samples and samples from humans suspected of liver fluke infections in Vietnam and Taiwan.

**Table 1:** Amino acid sequences of the various antigens used in these studies.

Antigen	Associated Species/Swiss Protein or GenBank Number	N-terminal Amino Acid Sequences
Recombinant Cs44	*C. sinensis* Q9UB18	Full protein sequence is shown, but recombinant protein included only amino acids 18–274 in brackets: MKFLKLVIIG ALFLNVLC **{**LD GGAQPPKSGD GGAQPPKSGD GGAQPPKSGD GGAQPPKSGD GGAQPPKSGD GGAQPPKSGD GGAQPPKSGD GGAQPPKSGD GGAQPPKSGD GGAQPPKSGD GGAQPPKSGD GGAQPPKSGD GGAQPPKSGD GGAQPPKSGD GGAQPPKSGD GGAQPPKSGD GGAQPPKSGD GGAQPPKSGD GGAQPPKSGD GGAQPPKSGD GGAQPPKSGD GGAQPPKSGD GGAQPPKSGA QRPFSHWIAG WFLVPLEVKA SDHF**}**
Recombinant Egg protein	*C. sinensis* Q81813_CLOSI	Full protein sequence is shown, but recombinant protein included only amino acids 17–253 in brackets: MKPICLLLVG LVSISL **{**TSGY KRGYNFGLED GRVATGRFYR GGYGDATGGE VSGYDYDLEG DLSASGSSAH AGRFGKQRHE EDDGFYTQGG SFYVSGKARR DDGYGITAGL KAKGNFYGTG TEGEGSQYEH VTTFRRGGGH DTKGKKKHYN EYDSYGQAKK YGDKKVANNF DLRGILKAKG KFDGYGKSDV SSEFEKYGKL GYSGSSKGYG GRDVYGKLKG KSEYDAYGKL KGYGSQNDYS KYGRHADYDA LGY**}**
Hepatocellular carcinoma-associated antigen 59	*O. viverrini* OON13732.1	MDPAKKRCKRTRRPSDSSES
Cathepsin B	*O. viverrini*ACT99884.1	MKRLFISYAILVFVNSFQDA
Cathepsin B2	*O. viverrini*ACT99885.1	MPWLILVFGTVLAAAGEVTG
Partial Cadherin Domain Protein	*O. viverrini* OON24061.1	RNECVEPQWKTLRFIHGYLD

## 2 Methods

### 2.1 Recombinant liver fluke protein and peptide targets

Recombinant partial *C. sinensis* adult protein Cs44 and *C. sinensis* egg protein were purchased from Bioclone Inc. (San Diego, CA, USA). Various *O. viverrini*-associated peptides were purchased from GenScipt Inc. (Piscataway, NJ, USA). The amino acid sequences of each antigen are shown in Table 1 below and can be validated by their Swiss protein or GenBank numbers given in Table 1.

### 2.2 Human serum samples

Ten anonymous human remnant serum samples from Taiwan and 10 anonymous human remnant serum samples from Vietnam from patients suspected of active or prior liver fluke infections were purchased from BioIVT Inc. (Westbury, NY, USA) and stored frozen at −70 °C until thawed for use. A pooled 100 ml normal human serum sample from unexposed American subjects was also purchased from SeraCare Life Sciences (Gaithersburg, MD, Cat. No. 1830-0002) and used for the NHS samples.

### 2.3 Other materials and instruments

A Dynex Opsys MR microplate reader (Chantilly, VA) was used to read ELISA results by absorbance at 405 nm. Flat bottom clear polystyrene 96-well plates were obtained from Greiner Bio-one GmBH (Austria). Immobilization buffer consisted of 0.1 M bicarbonate buffer (0.091 M sodium bicarbonate and 0.009 M of sodium carbonate, pH 9.2), 1X phosphate-buffered saline (PBS) and 5% Bovine Serum Albumin. “One-Component” ABTS (2,2′-Azino-bis(3-ethylbenzothiazoline-6-sulfonic acid) peroxidase substrate was obtained from SeraCare Life Sciences Inc.

### 2.4 ELISA procedure

Prior to antigen addition, 100 μl of bicarbonate immobilization buffer was dispensed per well and incubated at room temperature for 30 min to activate the wells for increased binding of the antigen. After discarding the buffer by inverting the plates in a waste container or a sink, plates were patted gently with wells side down on a dry paper towel to eliminate residual buffer. Protein and peptide antigens from Table 1 were used at a concentration of 1 μg/well in bicarbonate immobilization buffer (pH 9.2). Each plate included four replicate antigen wells and controls including wells without antigen or serum and absence of secondary antibody. The wells were then sealed and stored at 4 °C overnight. The next day, the plates were warmed to room temperature and the antigen solutions were aspirated. Wells were washed with 300 μl of 1X PBS. Plates were patted dry and wells were then blocked with 200 μl of 5% BSA dissolved in 1x PBS buffer for 2 h at room temperature. Human serum samples were thawed on ice, centrifuged at 10,000 rpm to remove any lipid layer, and the clear samples were diluted 1:10 with 1X PBS. After 2 h of incubation, the blocking solution was aspirated and excess blocker was removed by gently patting the plate with wells side down on a dry clean paper towel. On hundred μl of diluted serum samples were dispensed in their respective wells and wells were left at room temperature for 1 h. Serum samples were aspirated and wells were washed with 1X PBS as before but with gentle shaking for 30 s. PBS washes were repeated three times and after the last wash, excess PBS was removed by gently tapping the plate on a clean paper towel as described previously. One hundred microliters of horseradish peroxidase (HRP)-conjugated goat anti-human IgG secondary antibody (Invitrogen, Cat# A18817) was diluted 1: 1500 in 1X PBS and 100 μl of this diluted antibody-HRP conjugate was added to various wells. Plates were incubated for 1 h at room temperature. Plates were then washed 3 times with 1X PBS as described previously. After making sure that the wells are completely dry, 100 μl of ABTS peroxidase substrate (One-Component containing stabilized H_2_O_2_) was added to all wells. Plates were left at room temperature and incubated for 8 min with occasional and gentle shaking. Absorbance was then measured at 405 nm using the plate reader. Absorbance values were recorded in an Excel spread sheet and averages of four replicates with standard deviation values were determined. Calculation of p values from T-tests compared with NHS values indicated the presence or absence of liver fluke-reactive antibodies in the respective samples.

## 3 Results

A total of 20 serum samples collected from 10 individuals in Vietnam and 10 individuals in Taiwan suspected of active liver fluke infection were analyzed by indirect ELISA using recombinant antigens and specific peptides as specified in Table 1. Profiles of antibody levels for specific antigens tested are described in individual figures as shown below.

**Fig. 1:**
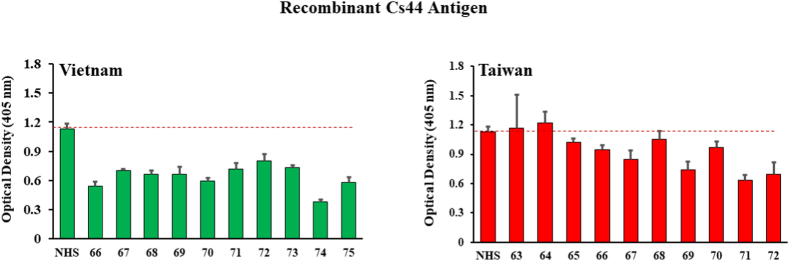
ELISA results for detection of human IgG against recombinant *C. sinensis* Cs44 antigen (amino acids 18–274) from ten human serum samples each drawn in Vietnam and Taiwan from patients suspected of present or past liver fluke infection. Bars represent the average optical density value of 4 independent wells with standard deviation error bars. NHS means normal human serum and numbers on the X-axis represent patient identification numbers assigned by BioIVT. ELISA results were considered positive if the OD value of the well with serum sample from an experimental group was higher with statistical significance than the OD value of the control (NHS) group.

Fig. 1 illustrates ELISA results using the recombinant *C. sinensis* Cs44 antigen from Bioclone as the target coated on microwells. One might expect to find some absorbance values well above the normal human serum (NHS) background among the 10 human serum samples for people suspected of liver fluke infections, but there were no significantly elevated values in either the Vietnam or Taiwan samples indicating that there were no significant IgG antibody levels against Cs44 in these samples.

**Fig. 2:**
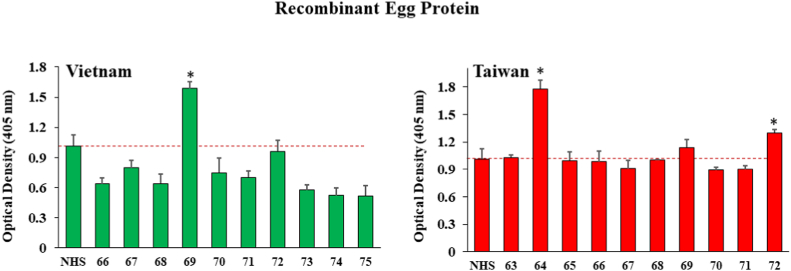
ELISA results for detection of human IgG against recombinant *C. sinensis* egg protein antigen (amino acids 17–253) from ten human serum samples each drawn in Vietnam and Taiwan from patients suspected of present or past liver fluke infection. Bars represent the average optical density value of 4 independent wells with standard deviation error bars. NHS means normal human serum and numbers on the X-axis represent patient identification numbers assigned by BioIVT.

Fig. 2 suggests that one of the Vietnam samples (sample ID# 69 with OD of 1.588 ±0.06) and two of the Taiwan samples (sample ID # 64 & 72 with OD values of 1.778 ± 0.09 and 1.2985 ±0.03 respectively) were significantly above normal human serum antibody levels (OD value of 1.015 ±0.11) when the *C. sinensis* recombinant egg protein was used as the target. This is interesting because one might not expect liver fluke eggs residing in the gut to induce IgG in serum, but the data suggest that this may have occurred.

**Fig. 3:**
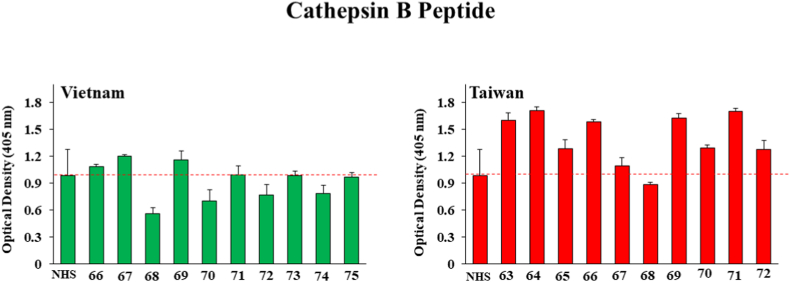
ELISA results for detection of human IgG against *O. viverrini* Cathepsin B antigen from ten human serum samples each drawn in Vietnam and Taiwan from patients suspected of present or past liver fluke infection. Bars represent the average optical density value of 4 independent wells with standard deviation error bars. NHS means normal human serum and numbers on the X-axis represent patient identification numbers assigned by BioIVT.

**Fig. 4:**
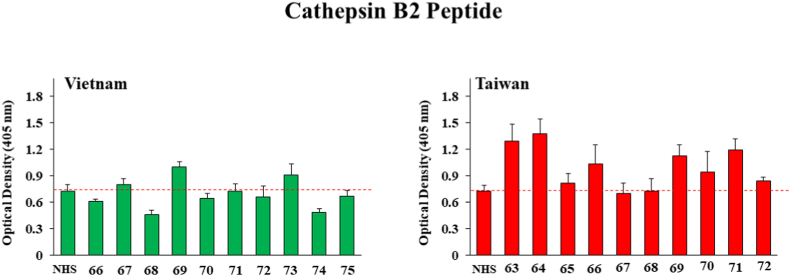
ELISA results for detection of human IgG against *O. viverrini* Cathepsin B2 antigen from ten human serum samples each drawn in Vietnam and Taiwan from patients suspected of present or past liver fluke infection. Bars represent the average optical density value of 4 independent wells with standard deviation error bars. NHS means normal human serum and numbers on the X-axis represent patient identification numbers assigned by BioIVT.

Fig. 3 shows that five of the Taiwan samples (samples with ID#s 63, 64, 66, 69 and 71) were statistically above the NHS antibody levels, with OD values of 1.6 ± 0.08, 1.7 ± 0.04, 1.58 ± 0.02, 1.629 ± 0.04, 1.7 ± 0.03 respectively but none of the Vietnam samples were above normal human serum antibody levels when *O. viverrini*-associated Cathepsin B was used as the target for serum antibody binding. A similar pattern was seen when using Cathepsin B2 peptide (shown in Fig. 4), although one of the Vietnam samples (ID# 68 with an OD value of 0.997 ± 0.05) was above background. Among the Taiwan samples, 63, 64, 66, 69, 71 and 72 showed significantly higher OD values (1.29 ± 0.19, 1.379 ± 0.165, 1.03 ± 0.21, 1.12 ± 0.12, 1.194 ± 0.12 and 0.844 ± 0.04 respectively compared to normal serum with a OD value of 0.7265.

**Fig. 5:**
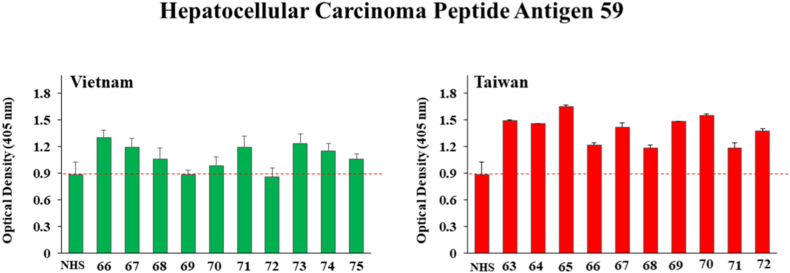
ELISA results for detection of human IgG against *O. viverrini* Hepatocellular Carcinoma Peptide Antigen 59 from ten human serum samples each drawn in Vietnam and Taiwan from patients suspected of present or past liver fluke infection. Bars represent the average optical density value of 4 independent wells with standard deviation error bars. NHS means normal human serum and numbers on the X-axis represent patient identification numbers assigned by BioIVT.

Fig. 5 suggests that there were numerous serum samples having IgG antibodies above normal serum levels against the Hepatocellular Carcinoma Peptide Antigen 59 in both the Vietnam and Taiwan regions. Among the Vietnam group, samples 74, 66, and 72 showed significantly higher OD values of 1.29 ± 0.088, 1.696 ± 0.09, and 1.235 ± 0.105 respectively compared to normal serum levels of 0.886 ± 0.13. From the Taiwan group, except #71 all other samples demonstrated higher OD values compared to OD levels of normal serum.

**Fig. 6:**
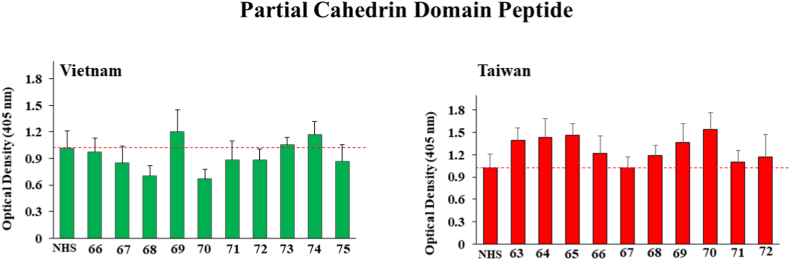
ELISA results for detection of human IgG against *O. viverrini* Partial Cahedrin Domain Peptide from ten human serum samples each drawn in Vietnam and Taiwan from patients suspected of present or past liver fluke infection. Bars represent the average optical density value of 4 independent wells with standard deviation error bars. NHS means normal human serum and numbers on the X-axis represent patient identification numbers assigned by BioIVT.

Finally, for Fig. 6, the data suggest slightly stronger immune responses for samples numbers 63, 64, 65 and 70 to the Partial Cahedrin Domain Peptide in the Taiwan group with OD values of 1.389 ± 0.16, 1.435 ± 0.25, 1.46 ± 0.159 and 1.53 ± 0.228 respectively while none of the samples were positive among the Vietnam group.

## 4 Discussion

The present report documents preliminary results intended to lead to novel liver fluke exposure ELISAs to detect human IgG reactive with several novel recombinant protein or N-terminal peptide targets from *C. sinensis* and *O. viverrini*. The data sets are admittedly limited to a mere ten human serum samples from patients suspected of having active or past liver fluke infections as determined by BioIVT. Thus, we cannot perform ROC (Receiver-Operating Characteristic) analysis or calculate percent sensitivity or specificity without firm medical diagnoses of liver fluke infection associated with each serum sample. Still, several Asian samples appeared to be statistically above the background from commercially available (SeraCare) pooled “normal” human serum (NHS) collected in the U.S.

Among the presumptive positive samples, several interesting patterns seemed to emerge. The first and most surprising result was that there were no seropositive samples reactive with recombinant Cs44 found from Vietnam or Taiwan. One might hypothesize that adult flukes living in human bile ducts would most certainly induce a strong immune response, but this was not supported by any of the Fig. 1 data. One possibility is that antibodies in the sera of actively or previously *C. sinensis*-infected patients might be reactive to the first 17 amino acids on the N-terminus of Cs44 which is deleted in the recombinant protein that was used (Table 1) or the reactive epitope is somehow hidden by immobilization on the surface of the microwells (i.e., missing or hidden epitope on the recombinant Cs44).

It was also somewhat surprising that some human sera were positive for antibodies against the *C. sinensis* recombinant egg protein in both Vietnam and Taiwan as shown in Fig. 2, because the eggs are presumably sequestered away from the blood stream in the gut lumen. However, gut-associated lymphoid tissue (GALT) in the intestinal wall as well as mingling of peripheral blood with any potential breaks in the gut lining may have led to IgG antibodies emerging in the blood serum.

Both of the Cathepsin B (B and B2) peptides which are thought to be secreted appeared to give stronger positive responses from patients in Taiwan than those in Vietnam as illustrated in Fig. 3, Fig. 4. Hepatocellular Carcinoma Peptide Antigen 59 elicited perhaps the strongest and most frequent responses in both Vietnam and Taiwan as demonstrated by Fig. 5 and Partial Cahedrin Domain Peptide may be showing a slightly stronger response from Taiwanese vs. Vietnamese samples, although the data are similar from both regions.

The present work represents preliminary screening of several novel peptides and recombinant proteins for potential use as liver fluke exposure antigens in future ELISAs. Sample sizes were limited to ten serum samples per country due to the extreme difficulty in obtaining such excess archival human serum samples. Thus, no solid conclusions can be drawn. Yet, the limited data appear to suggest that some of the novel recombinant protein and peptide antigens selected and tested herein are promising and warrant further study as possible targets for detecting antibodies by ELISA from humans suspected of liver fluke infections.

## Funding

Work was funded by the U.S. National Cancer Institute (NCI) Small Business Innovative Research (SBIR) Contract No. 75N91019C00050.

## Author's contributions

Sumathy Mohan and Mohan Natarajan conducted the ELISA tests and compiled the data. John G. Bruno conceived of the project and wrote the manuscript.

## Declaration of competing interest

There are no competing interests for all authors.

## Data Availability

Data will be made available on request.
